# “Photobiomics”: Can Light, Including Photobiomodulation, Alter the Microbiome?

**DOI:** 10.1089/photob.2019.4628

**Published:** 2019-11-12

**Authors:** Ann Liebert, Brian Bicknell, Daniel M. Johnstone, Luke C. Gordon, Hosen Kiat, Michael R. Hamblin

**Affiliations:** ^1^ Australasian Research Institute, Wahroonga, Australia.; ^2^ Department of Medicine, University of Sydney, Camperdown, Australia.; ^3^ Faculty of Health Sciences, Australian Catholic University, North Sydney, Australia.; ^4^ Discipline of Physiology, University of Sydney, Camperdown, Australia.; ^5^ Faculty of Medicine and Health Sciences, Macquarie University, Marsfield, Australia.; ^6^ Faculty of Medicine, University of New South Wales, Kensington, Australia.; ^7^ Wellman Center for Photomedicine, Massachusetts General Hospital, Boston, Massachusetts.; ^8^ Department of Dermatology, Harvard Medical School, Boston, Massachusetts.; ^9^ Harvard-MIT Division of Health Sciences and Technology, Cambridge, Massachusetts.

**Keywords:** photobiomodulation, microbiome, bacteria, metabolome

## Abstract

***Objective*:** The objective of this review is to consider the dual effects of microbiome and photobiomodulation (PBM) on human health and to suggest a relationship between these two as a novel mechanism.

***Background*:** PBM describes the use of low levels of visible or near-infrared (NIR) light to heal and stimulate tissue, and to relieve pain and inflammation. In recent years, PBM has been applied to the head as an investigative approach to treat diverse brain diseases such as stroke, traumatic brain injury (TBI), Alzheimer's and Parkinson's diseases, and psychiatric disorders. Also, in recent years, increasing attention has been paid to the total microbial population that colonizes the human body, chiefly in the gut and the mouth, called the microbiome. It is known that the composition and health of the gut microbiome affects many diseases related to metabolism, obesity, cardiovascular disorders, autoimmunity, and even brain disorders.

***Materials and methods*:** A literature search was conducted for published reports on the effect of light on the microbiome.

***Results*:** Recent work by our research group has demonstrated that PBM (red and NIR light) delivered to the abdomen in mice, can alter the gut microbiome in a potentially beneficial way. This has also now been demonstrated in human subjects.

***Conclusions*:** In consideration of the known effects of PBM on metabolomics, and the now demonstrated effects of PBM on the microbiome, as well as other effects of light on the microbiome, including modulating circadian rhythms, the present perspective introduces a new term “photobiomics” and looks forward to the application of PBM to influence the microbiome in humans. Some mechanisms by which this phenomenon might occur are considered.

## Introduction to Light Therapy and the Microbiome

Light is known to have wide-ranging effects in multiple biological kingdoms,^1^ and has been used for many years as a therapeutic agent, although in recent years (in the modern era of pharmaceuticals) it has fallen from favor. Finsen received the Nobel Prize for Physiology or Medicine in 1903 for his work in treating cutaneous tuberculosis with UV light and smallpox with red light.^2^ Bright light therapy (phototherapy) is still the first-line therapy for seasonal affective disorder, for circadian rhythm misalignment, and is used for sleep disorders (including for Parkinson's disease^3^) and Alzheimer's disease at the Mayo clinic in the United States (https://www.mayoclinic.org/tests-procedures/light-therapy/about/pac-20384604). Bright light therapy has been suggested as a therapy for depression and other neuropsychiatric conditions^4^ and is currently under trial as a therapy for Parkinson's disease.^5^ Neonatal hyperbilirubinemia has been routinely treated since the 1970s with blue light. Red light for the treatment of retinopathy of prematurity, caused by oxygen toxicity, is now being trialed,^6–8^ and could also be tested for methanol-induced retinal damage, diabetic retinopathy^7^ and age-related macular degeneration,^9–11^ and cognition.^12^ It is becoming increasingly apparent that daylight and circadian rhythms play an important part in many treatments. For example, the timing of therapy (chronotherapy) in cardiovascular disease influences therapeutic success^13^ and the position (sunny versus dull) of the patients in cardiac intensive care units who are recovering from myocardial infarction, influences their mortality and length of hospital stay.^14^

It has been recognized in recent years that the gut microbiome is inextricably linked with health and disease. The gut microbiome (whether healthy or not) has a profound effect on inflammation and cytokine production, production of metabolites, and direct vagal nerve stimulation. It is also recognized that there is a complex communication between the body and the various microbiomes within the body. It is the contention of the authors that light, and specifically photobiomodulation (PBM), can alter the microbiome, possibly through this communication.

Light can affect the microbiome indirectly through the daily circadian rhythm. The metabolome is intimately associated with chronobiology and hence with ambient light,^13^ with the circadian clock regulating levels of metabolites, including those from the microbiome, which in turn can affect metabolome.^15^ The effect of the circadian rhythm on the microbiome has been demonstrated^16–18^ and the bacteria responsible for decreased gut integrity and increased lipopolysaccharide transport are upregulated in mice after disruption of the sleep/wake cycle.^18^ In addition to circadian rhythm, light also has an indirect effect on the microbiome through vitamin D, produced by the action of sunlight on keratinocytes. Vitamin D is known to boost immune function by the induction of antimicrobial peptide genes and the regulation of tight junction proteins in the epithelial layer of the intestine^19,20^ and to maintain microbiome homeostasis and protect against colitis in mice,^21^ possibly by controlling inflammation.^22^ Vitamin D deficiency has been linked with such conditions as irritable bowel disease, obesity and diabetes, proinflammatory cytokines, intestinal barrier disturbance,^23^ and gut dysbiosis,^22^ and has been suggested to influence immune-mediated disease.^24^ Similarly, contaminants in food, such as fertilizers, pesticides, and herbicides, can have their toxicity increased by sunlight,^25^ which may also have an adverse effect on the microbiome.

It is also apparent that blue light-emitting diode (LED) screens and lights used at night can suppress melatonin secretion and affect circadian rhythms with consequent effects on health^26^ and it has also been demonstrated that red light (morning light) in humans can influence both leptin and ghrelin concentrations,^27^ which play a role in energy homeostasis, hunger, and satiety. Recently, Basha and colleagues^28^ have shown that fluorescent lighting can affect the oxidative stress of rats and Boswell and colleagues have shown that fluorescent lighting can have effects on gene regulation and inflammatory processes,^29^ which have the potential to affect the microbiome.

We introduce the term “photobiomics” to characterize the combined effects of light (PBM or otherwise) on metabolomic factors, the microbiome, and the interaction between the two.

## Photobiomodulation

Light therapy was, in a sense, rediscovered by Mester et al. who found that low-power laser light had a positive effect on wound healing and hair regrowth in mice.^30^ PBM is the newly adopted consensus term to describe the therapeutic application of low levels of red and/or near-infrared (NIR) light to treat a multitude of different diseases and disorders. PBM used to be known as “low-level laser or light therapy,” but the name was changed to reflect the increasing use of LEDs, the possibility of inhibition as well as stimulation, and to avoid the undefined nature of the term “low level”.^31^

The mechanisms of action of PBM have been widely investigated in recent years, and additional mechanistic information is still being discovered. Nevertheless, it is generally accepted that the single most important chromophore in the red and NIR regions of the spectrum is cytochrome c oxidase (CCO), which is unit IV of the mitochondrial respiratory chain. When CCO absorbs light, the enzyme activity is increased leading to increased electron transport, more oxygen consumption, higher mitochondrial membrane potential, and increased ATP production.^32^ Signaling molecules are produced, including a brief burst of reactive oxygen species (ROS), nitric oxide, cyclic AMP, and movements in intracellular calcium. These signaling molecules result in activation of a host of transcription factors, and changes in the expression of a multitude of gene products, including structural proteins, enzymes, and mediators of cell division and cell migration.^32^ Interestingly, a recent report has thrown into question the central role of CCO in the mechanism of PBM action.^33^

In addition to the proposed action of PBM on CCO, and the consequent signaling, other mechanisms operate at a cellular and tissue level, including nonvisual phototransduction cascades involving opsins (OPN 1–5). Recent evidence has shown that blue (415 nm) and green (540 nm) light are absorbed by opsins that then trigger opening of transient receptor potential (TRP) calcium ion channels.^34^ The interaction between PBM and opsins in the skin has been reviewed by Khan and Arany.^35^ There is recent evidence that melanopsin (OPN4), found in the eye, is also present in adipocytes,^36,37^ throughout the brain,^38^ in skin cells, and blood vessels.^39^ Melanopsin is a tri-stable switch that can absorb in the red spectrum.^40^ Light penetrating the skull (sunlight and PBM) can alter melanopsin.^41^ The 380 nm light is also absorbed by neuropsin (OPN5) in the skin, retina, and nervous system and light is absorbed in hair follicles by OPN2 and OPN3. Absorption by opsins results in downstream nonvisual phototransduction cascades, which in turn presumably influence protein conformation at the cell membrane and possibly cytoskeleton modulation cascades.^42^

Santana-Blank and Rodrıguez-Santana^43,44^ have argued that the structure of water and its absorption of NIR is integral to PBM mechanisms. PBM, delivered as low-level laser, has been shown to have a dramatic effect on the cytoskeleton structure of nerve cells, with the rapid formation of transient varicosities, and consequent nerve blockade for pain relief.^45–49^ PBM also affects the cytoskeleton of other cells besides neurons, such as epithelial cells,^50^ keratinocytes,^51^ and fibroblasts,^52^ and has been shown to have effects on protein conformation, including calcium ion channels.^53–55^ PBM also has an effect on brain oscillation patterns, with changes to alpha, beta, gamma, delta, and theta waves in both mice^56^ and humans^57^ (El Khoury, et al., unpublished observations) and effects on the default mode network.^58^

**FIG. 1.**
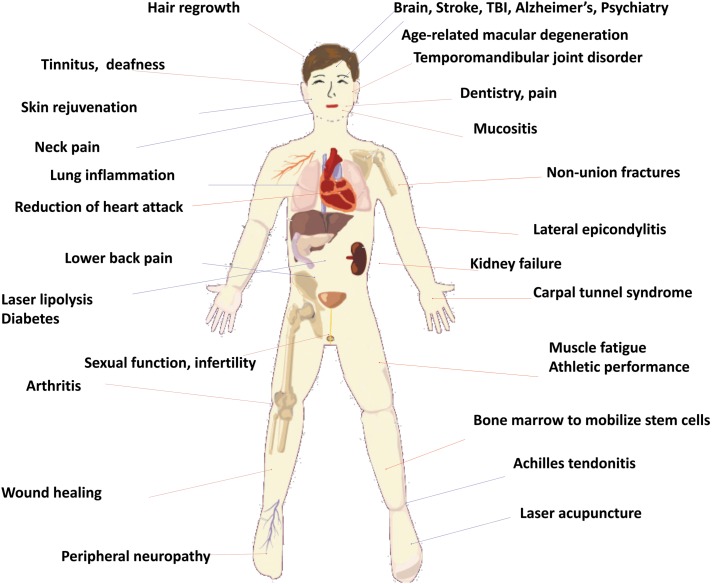
Schematic illustration of the wide variety of human diseases and disorders that have been treated by PBM. PBM, photobiomodulation.

Many of the diseases treated by PBM are localized by nature and include orthopedic conditions, such as inflammation in joints and tendons, wounds, and fractures. In these applications, light is usually delivered as a spot (often from a focused laser beam) onto the affected area of tissue. The wavelengths employed are mostly in the red regions (630–680 nm) or in the NIR region (780–940 nm), although longer wavelengths (980 and 1064 nm) have also shown benefit.^32^ Power densities are usually in the region of 10–100 mW/cm^2^, together with energy densities in the region of 4–50 J/cm^2^. It is usual to use higher power densities and higher energy densities to treat lesions that are located deeper in the tissue, such as large joints, spine, and brain. Figure 1 gives a diagrammatic illustration of many disorders that have been treated by PBM.

In recent years there has been accumulating evidence that there are also significant systemic effects produced by PBM, whereby application of PBM to one part of the body promotes beneficial outcomes in remote tissues. The exact mechanisms underlying these systemic effects of PBM are not completely understood, but some hypotheses have been put forward. One such involves the absorption of light by muscle. The large mass of muscle that is exposed to light combined with the high numbers of mitochondria inside muscle cells, means that the resulting increase in metabolism can have effects on the whole body. Not only has PBM been shown to be important for increasing athletic performance, and encouraging recovery from strenuous exercise,^59^ but also the increased consumption of glucose, and the burning of fat goes some way to improving diabetes, metabolic syndrome, and countering obesity and systemic inflammation.^60^ In fact, the study of PBM and metabolomics is a nascent area of study.^61^ The use of PBM to treat cardiovascular diseases, such as hypertension, high cholesterol, disorders of circulation, clotting disorders is a still-emerging field.^62^

Given its important effects on mitochondria within cells that are irradiated, PBM may trigger a signaling system between mitochondria in peripheral cells and cells residing in the brain,^63^ facilitated by an unknown mediator termed a “mitokine”. In *Caenorhabditis elegans* it has been shown that mitochondrial perturbations in one tissue type initiate a mitochondrial stress response in distal, seemingly unaffected tissues.^64,65^ Aside from the effects on mitochondria, PBM is also known to induce proliferation and migration of stem cells.^32,66–69^ It has been suggested that through this induction, PBM can mobilize stem cells that home specifically to damaged tissue to aid in repair. For example, in animal models, PBM applied to the tibia results in migration of stem cells and mitigation of damage in models of myocardial infarction^70–72^ and Alzheimer's disease.^73,74^ Another possibility is that PBM treatment could remediate mitochondrial dysfunction in gut neurons, reinstating the complex bidirectional communication system between the enteric nervous system and the central nervous system (the gut/brain axis). This may have particular significance for neurodegenerative conditions, such as Alzheimer's and Parkinson's diseases, both of which involve early pathological abnormalities in the gut/brain axis.^75–79^

PBM has a pronounced effect on inflammatory processes by reducing oxidative stress, reducing proinflammatory cytokines, and changing macrophage phenotype.^80^ The local effect of PBM on inflammatory pathways most probably has systemic consequences. It is possible that circulating immune cells (mast cells, macrophages, etc.), stimulated by PBM,^81–84^ could transduce protective signals from distal tissues to sites of injury such as the brain, heart, or gut.

## Human Microbiome

The human microbiome comprises many billions of bacteria, archaea, protists and viruses that live in close association with our bodies. There is a microbiome associated with our skins, with our mouth and nose, with our ears and eyes, with our respiratory tracts, our urogenital tracts, and with our gut. Over the past few years, there has been increasing interest in the interaction between our microbiome and the cells and tissues of our body. The gut microbiome contains somewhere in excess of 10^14^ bacteria, representing over 1000 species (upward of 6000 strains) and contributing 150 times the genetic material of our own genome.^85^ It has become evident that the gut microbiome communicates with our body and that our body in turn communicates with the microbiome.^86,87^ For these reasons, the gut microbiome is often given the importance of an additional organ and, in common with other organs, has its own circadian rhythm.^88^ The intimate relationship of the human host and the bacteria of the microbiome is often referred to as the holobiont, with the holobiome being the total genetic material of the partners.

The gut microbiome of lean and healthy humans (and model organisms) is quite different to that of obese humans and animals. In fact, it is now recognized that a healthy gut microbiome is to a large extent responsible for a healthy individual. Changes in the health status of humans and model organisms are accompanied by changes in the gut microbiome, which can include genus-level, family-level, and even phylum-level fluctuations in the microorganisms that are present, as well as changes in microbial diversity (either increased or decreased diversity). The microbiome shows differences with different metabolic diseases and disorders and there is a microbiome component to such disparate diseases as cardiovascular disease (including heart failure) and neurological disorders (including Parkinson's disease).^89–91^ Thus, it has been recognized that there is a microbiome/gut/brain axis, a microbiome/gut/heart axis, and possibly a microbiome/gut/muscle axis,^92^ a microbiome/gut/lung axis,^93^ and a microbiome/gut/skin axis.^94^ More recently, links between the gut and pain,^95^ the gut and arthritis, and the gut and neutrophils^96^ have been proposed.

The composition of the microbiome is affected by birthing practice (vaginal/cesarean), growth through infancy (breast milk/formula) to adulthood (vegan/meat-based diet), genetics (the HLA-B27 gene may cause gut dysbiosis, leading to spondylarthrosis^97^), aging, stress, antibiotics, and diet (including alcohol consumption, prebiotics, probiotics), all of which shape the overall composition of the microbiome.^87^ A changed diet (e.g., high fat, high sugar, plant-based, vegan, etc.) can change the microbiome in the short or long term.^90,98^ A diet high in a diversity of plant products is generally linked with greater species richness in the gut,^99^ whereas a more meat-based diet leads to a replacement of carbohydrate-fermenting bacteria with bile-tolerant bacteria.^86^

Gut microbiota assist in food digestion, change the kilojoule yield of the food, and contribute to vitamin and mineral production and intake, and more efficient energy production from a dysregulated microbiome may be one factor in obesity,^90,100^ due to increased energy harvest from polysaccharides and inhibition of fasting-induced adipose factor and monophosphate-activated protein kinase, both of which influence deposition of body fat.^101^ A western diet results in changes in the microbiome of both humans^98^ and mice,^102^ a trait which is transferable with fecal transplants.^90^ Antibiotics as well as nonantibiotic drugs, including proton pump inhibitors, can disrupt the microbiome and generate long-term effects.^86,103,104^ Metformin and Acarbose, both used to treat type 2 diabetes, have been shown to have positive effects on the microbiome.^105–107^

**FIG. 2.**
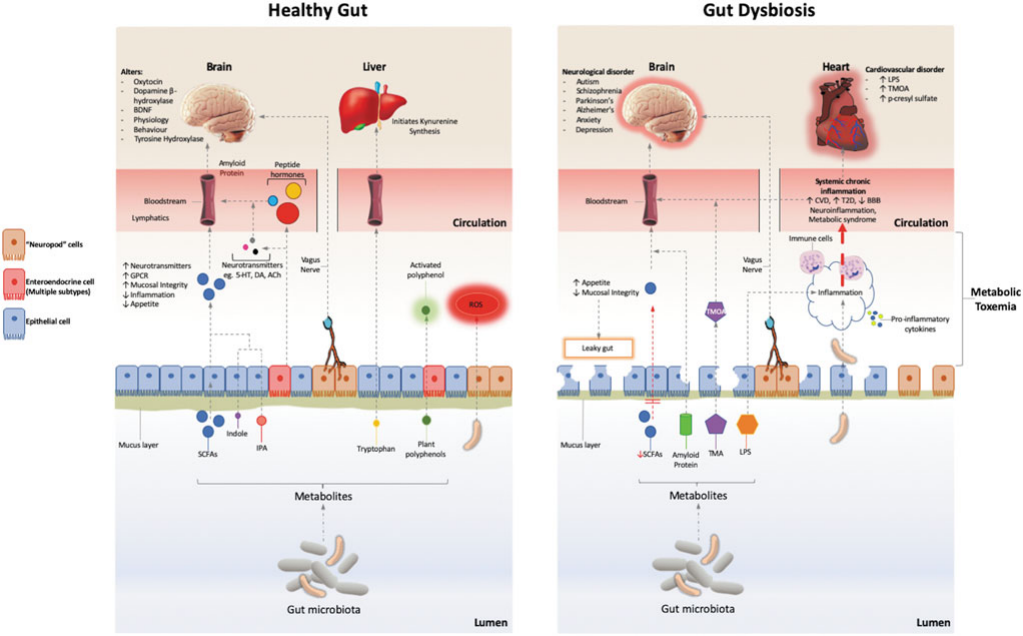
The main interactions between the microbiome and the body with a healthy and unhealthy microbiome. Bacteria in the microbiota produce SCFA, influence redox signaling, influence tryptophan metabolism, activate plant polyphenols, and produce neurotransmitters, hormones, and peptides. This has the effect of promoting a thick mucus layer, an intact epithelium, and producing numerous downstream metabolomic effects. The microbiome communicates with the body through these metabolites as well as direct communication through the vagus nerve. In dysbiosis, reduced SCFA levels weaken epithelial integrity, increasing efflux of bacterial metabolites, such as proteins and LPS, contributing to an increased inflammatory response and “leaky gut.” Production of TMOA and p-cresyl sulfate are correlated with cardiovascular disease. 5-HT, serotonin; ACh, acetyl choline; BBB, blood brain barrier; CVD, cardiovascular disease; DA, dopamine; GPCR, G protein couple receptor; IPA, indolepropionic acid; LPS, lipopolysaccharide; ROS, reactive oxygen species; SCFAs, short chain fatty acids; T2D, type II diabetes; TMA, trimethylamine; TMOA, trimethylamine oxide.

The main communication pathways between the microbiome and the body are through the immune response, redox signaling, the endocrine system and the enteric/vagus nerve pathway; summarized in Fig. 2. One of the major known effects of the microbiome is the release of short-chain fatty acids (SCFAs), such as butyrate, acetate, and propionate, produced by fermentation of undigested polysaccharides or proteins. SCFAs influence the integrity of the gut mucosa by increasing epithelial integrity and production of mucus and influence the body's energy balance, inflammatory response, and protect against cancer.^89,90,108^ They are potent signaling molecules, affecting a number of G-protein-coupled receptors, resulting in such effects as increased glucagon-like peptide 1, leptin, and peptide YY; increased insulin sensitivity; increased energy expenditure; increased satiety; and protection against irritable bowel disease and cancer (reviewed by Koh, et al.^108^). The microbiome also has a role in tryptophan metabolism, producing tryptophan catabolites such as indole and indolepropionic acid (IPA),^109^ which are also anti-inflammatory and influence the kynurenine pathway.^110^ Gut bacteria (especially the lactobacilli) are known to generate ROS, at levels that are able to influence cell signaling and reduce the inflammatory response.^111^ The microbiota in the gut also produce the active forms of polyphenols, by altering the bound state of these molecules in plant foods.^112^

Microbes in the gut produce other metabolites, including neurotransmitters and hormones, which cross the intestinal mucosa, interact with cells and tissues of the body, and contribute to the metabolites that can be detected in the circulation.^113^ These signaling molecules may be identical to those produced by the body or close analogs that the body can recognize and have a role in regulating appetite, weight gain, insulin sensitivity, peripheral lipid storage, and liver and muscle energy balance. Such signaling molecules include catecholamines, serotonin, gamma aminobutyric acid (GABA), dopamine, acetylcholine, α-MSH, norepinephrine, and melatonin, all of which can leave the gut lumen.^109,114,115^ The microbiota also influences the production of metabolites by the enteroendocrine cells. For example, 90% of the body's serotonin is produced by enteroendocrine cells in the gut,^116^ which has a major influence on mood and cognition.^117^

Communication between the microbiome and the brain (microbiome/gut/brain axis) is also possible through the vagus nerve, the direct link between the brain and the enteric nervous system, which communicates directly with the gut lumen and is exposed to microbially produced neurotransmitters.^118^ Recently, enteroendocrine cells (so-called “neuropod” cells) have been shown to synapse with the vagus nerve to transmit signals directly from the gut to the brain in a single synapse.^119^ The vagus nerve can influence gut motility and mucin secretion, both of which will affect the microbiome.^113^ In addition, SCFA have also been shown to directly influence the sympathetic nervous system, through G-protein-coupled receptors.^120^

## Microbiome and Human Disease

A dysregulated microbiome having an effect on host health is known as dysbiosis. This can be caused by stress, aging, antibiotics, hygiene breakdown, and diet (absence of fiber and resistant starches). Dysbiosis will lead to decreased mucosal integrity and the movement of bacteria and microbial products into the portal circulation, the liver, and the systemic circulation (Fig. 2). These products include lactic acid, ammonium ions, endotoxins, bacterial cell wall components (lipopolysaccharide and peptidoglycan), membrane lipids, DNA, and whole bacterial cells. An altered microbiome can affect lipid metabolism, glucose metabolism, protein turnover, and redox balance as well as increasing biomarkers such as cholesterol, free fatty acids, fibroblast growth factor 21, bilirubin and lactate,^121^ and inflammatory markers such as interleukin 6 (IL-6) and tumor necrosis factor alpha (TNF-α).

The reduced SCFA production by the microbiome due to dysbiosis leads to decreased mucosal integrity and a thinning of the mucous layer.^122^ The leaking of microbial metabolites, products, or entire microbes from the intestinal lumen into the tissues sets up an inflammatory response, which then further reduces the integrity of the gut, leading to systemic chronic inflammation.^123^ Cytokines produced by the mucosal immune system can be released into the gut lumen and so in turn affect the microbiome. The inflammatory response associated with dysbiosis is correlated with obesity, ulcerative colitis, irritable bowel disease and Crohn's disease, metabolic syndrome, type 2 diabetes, cardiovascular disease, and cancer.^91,101,108,122,124–127^ Inflammation will also directly affect the central nervous system, through the vagus nerve, the sympathetic and parasympathetic nervous systems, and the neuroimmune system.^128^ The systemic and neuroinflammation associated with dysbiosis has been associated with cardiovascular disease (including hypertension, atherosclerosis, and heart failure), the mild cognitive impairment of aging, and a number of intractable neurodegenerative diseases and neurological disorders, including multiple sclerosis, Alzheimer's disease (gut and oral microbiome), Huntington's disease, autism spectrum disorder, schizophrenia, anxiety, and depression in both humans and laboratory rodents.^129–134^

Dysbiosis also disrupts tryptophan metabolism, shifting the balance of serotonin and kynurenine pathways.^135^ A disturbed kynurenine pathway has been linked to Parkinson's,^136^ cardiovascular disease,^137^ multiple sclerosis, amyotrophic lateral sclerosis (ALS), and other neurological diseases.^138^

There is a particularly strong link between the microbiome and Parkinson's disease, where the constipation suffered by a majority of Parkinson's disease sufferers^139^ is linked to α-synuclein accumulation in the enteric nervous system, increased intestinal permeability (leaky gut), and local inflammation (increased proinflammatory cytokines), which can occur years before the neural symptoms of Parkinson's disease become apparent.^140^ Interestingly, both rats and transgenic *C. elegans* models of Parkinson's disease show increased aggregation of α-synuclein as well as increased neural inflammation when exposed to bacteria that produce “curli”, a bacterial amyloid protein.^141^

The intestinal microbiome appears to have a causal role in the development and progression of atherosclerosis. In addition to obesity being a major risk factor for cardiovascular disease, dysbiosis results in the production of trimethylamine oxide produced in the liver from trimethylamine released by the gut microbiome.^142^ Trimethylamine oxide is a predictor of cardiovascular disease, although the causative link has yet to be established.^143,144^ Atherosclerotic cardiovascular disease also appears to be correlated with distinctive differences in the microbiome, including increased abundance of *Enterobacteriaceae* and *Streptococcus* spp.^145^ Dysbiosis has been linked with hypertension, atherosclerosis, arterial thrombosis, altered cholesterol and lipid profile, and heart failure^146^ and the gut microbiome has been shown to have a direct role in regulating blood pressure^144^ and blood lipids^147^ in rodents. Additionally, there is also a major link between the oral microbiome and cardiovascular disease, with *Porphyromonas gingivalis* (the causative agent in oral gingivitis) linked to atherosclerosis and found in the atherosclerotic plaques.^148^

It has also become apparent that dysbiosis is associated with chronic pain syndromes, including visceral pain,^149^ migraine (gut and oral microbiomes),^150^ chronic prostatitis and pelvic pain (gut and urogenital microbiomes),^151^ and autoimmune diseases such as rheumatoid arthritis.^152^

## PBM Alters the Microbiome

**FIG. 3.**
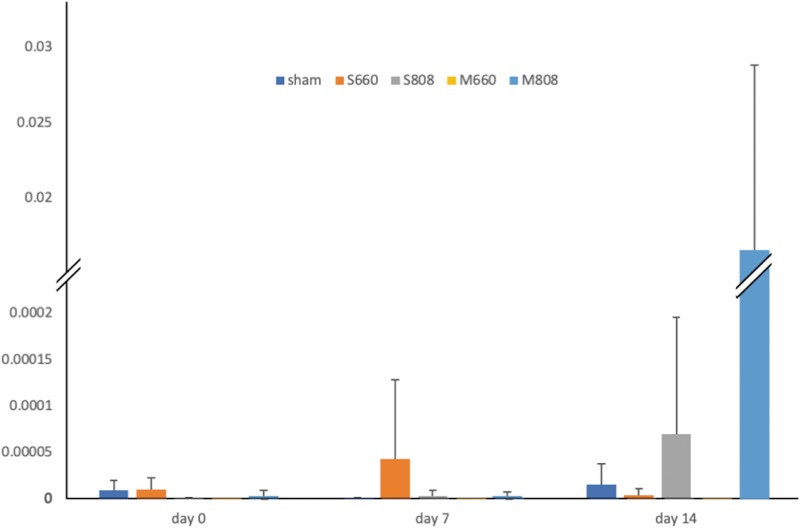
Change in the proportion of *Allobaculum* sp. in the total microbiota after PBM treatment with red and infrared laser. M, multiple (three times per week/2 weeks) dose of PBM; S, single dose of PBM. (adapted from Bicknell et al.^153^).

We have shown in a previous study^153^ that PBM, delivered as low-level laser, to the abdomen of healthy mice can produce a significant change in the gut microbiome. PBM significantly altered the microbial diversity of the microbiome, an effect most pronounced in mice treated three times per week with NIR light (808 nm), but not apparent with a single treatment with red light. PBM also produced a 10,000-fold increase in the proportion of the beneficial bacterium *Allobaculum* in the microbiota of mice after 14 days of treatment with NIR light but not with red light (Fig. 3).

This study has recently been repeated (unpublished) with larger numbers of mice in the experimental groups (10 per treatment group). The wavelength was again shown to be an important parameter, with NIR wavelengths showing a more pronounced effect than red light, and the proportion of bacteria associated with a healthy microbiome in mice generally increased while the proportion of bacteria associated with a dysregulated microbiome generally decreased. Blivet and colleagues have also hypothesized that the microbiome (in mice) is important for the treatment of Alzheimer's disease with PBM^154^ and have shown significant changes in the microbiome of mice injected with β-amyloid after treatment with a combination of PBM wavelengths and a static magnetic field (personal communication and^155^). Recent preliminary work from our laboratory (unpublished) has also indicated that changes in the human (quasimetabolic syndrome) microbiome occur after treatment with PBM, including increases in *Akkermansia muciniphila*, *Bifidobacterium* sp., and *Faecalibacterium* sp., all recognized as correlated with a healthy microbiome,^156–158^ and decreases in the Firmicutes:Bacteroides ratio, proposed as an indicator of gut health.^159,160^

UV therapy of skin has been shown to affect the skin microbiome by altering barrier function, leading to microbial-specific skin-resident memory T cells, disrupting the healthy balance between skin microbiome and skin immune cells, and resulting in chronic inflammation and diseased skin.^161^ On the other hand, UV irradiation of blood has been used for infections, autoimmune diseases, and some metabolic disorders.^162,163^ The mechanisms of action are still uncertain despite many years of investigation.

## Mechanisms of Action of PBM on the Microbiome

Because the whole field of “photobiomics” is so new, the discussion of possible mechanisms of action must remain highly speculative. They key question that must be addressed by further research is whether the light is primarily absorbed by the microbial cells themselves that make up the human microbiome, by the host cells that surround the microbes (or indeed cells that are distant from them), or by a combination of both microbes and host cells. The known chromophores for PBM, such as CCO, opsins, and flavoproteins, have mainly been investigated in mammalian cells. Nevertheless, there is a considerable body of work, largely emanating from Tiina Karu in Russia that a diverse range of bacterial species (both Gram-positives and Gram-negatives) and fungal (including yeast) cells do indeed respond to PBM.^164,165^ This response was mostly shown by increased proliferation of the microbial cells, but considering the biphasic nature of the PBM dose/response curve,^166,167^ at higher doses, inhibition was also observed. Similarly, de Sousa and colleagues^168^ have also shown that PBM inhibits the *in vitro* growth of bacteria that infect skin ulcers.

Alternatively, the alteration of the microbiome that was observed in the mouse experiments may be due to a secondary effect of PBM, affecting the mouse inflammatory response, and in turn affecting the gut microbiota. This is entirely feasible, given the intimate relationship between the microbiome (healthy and dysbiosis) and the inflammatory response. It is hypothesized that this effect may be due to the well-known anti-inflammatory and redox signaling effect of PBM.^60,80^ PBM can reduce proinflammatory cytokines, such as IL-6, TNF-α, IFN-γ,^169^ and change the activity of macrophages and neutrophils.^80^ Importantly PBM can change the “polarization state” of cells from macrophage lineage^170^ proinflammatory M1 to anti-inflammatory M2 phenotype.

In a series of experiments on Parkinson's disease, Stone, Johnstone, Mitrofanis and colleagues have shown that neuroprotection against Parkinsonian MPTP insult (in mice) can be achieved with PBM delivered to areas of the body remote from the brain.^36,171–174^ This abscopal effect of PBM is postulated to be due to immune cells, stem cells, or a circulating (unidentified) mediator. The possibility exists that this mediator is linked to changes in the microbiome.

## Potential Applications to Human Disease

It is entirely possible that some of the beneficial effects of PBM on systemic conditions and metabolic disorders that have historically been observed have been due to effects on the gut microbiome rather than the local tissue and this possibility has gone unrecognized until now. The lack of convincing scientific mechanistic evidence obtained so far, for the well-established abscopal effects of PBM, for instance, those seen in animal models of Parkinson's disease and in some cardiovascular disorders, suggests there may be room for this alternative explanation.

PBM may serve as a way to beneficially change the microbiome for a number of different inflammatory and neurological diseases (such as cardiovascular and Parkinson's diseases). The obvious approaches to try to improve the microbiome in humans such as diet, probiotics, and fecal transplants have had some success, but these treatments do not amount to a complete solution for the entire problem. Fecal transplants are currently being used for *Clostridium difficile* infection, irritable bowel disease, ulcerative colitis,^175^ and are also being considered for some nonintestinal metabolic diseases. Fecal transplants have been shown (in mice) to suppress neuroinflammation and TNF-α signaling, and to reduce the symptoms of Parkinson's disease and dysbiosis.^176^ PBM has the potential to act as an adjunct treatment (along with modifications of diet and exercise) to rebalance the microbiome. A healthy microbiome would balance SCFA production, serotonin/kynurenine pathways, trimethylamine metabolism, and dopamine and neurotransmitter production, which, in turn may affect the outcome of some of the most difficult-to-treat diseases, including Parkinson's disease, multiple sclerosis, amyotrophic lateral sclerosis, attention-deficit/hyperactivity disorder, and autism spectrum disorder.

## Conclusions

While metabolomics specifically excludes the microbiome, the two are inexorably linked; the microbiome directly affects the body and the body also influences the microbiome. The combination of the metabolome and the microbiome (the holobiont or holobiome) appears to be able to be changed by light, specifically by PBM. In light of the evidence that PBM can influence the microbiome and the known effect of PBM on cytokines, transcription factors, and the metabolome we introduce the term “*photobiomics*” to represent the combined effects of PBM on metabolomic factors, the microbiome, and the interaction between the two. Photobiomics most probably has a wider application than simply PBM. As is now generally understood, light has an effect on a wide range of living organisms in multiple biological kingdoms.^1^ Light in general may affect the microbiome as a downstream effect. The microbiome has been increasingly shown over the last decade to be a powerful influence on a range of diseases and to be very important in the maintenance of optimum health. The ability of PBM to influence the microbiome (if proven to be applicable to humans) will allow an additional therapeutic route to target multiple diseases, including cardiovascular disease and Parkinson's disease, many of which have thus far eluded effective treatment approaches.
